# Parabens Adsorption onto Activated Carbon: Relation with Chemical and Structural Properties

**DOI:** 10.3390/molecules24234313

**Published:** 2019-11-26

**Authors:** Astrid Roxanna Moreno-Marenco, Liliana Giraldo, Juan Carlos Moreno-Piraján

**Affiliations:** 1 Departamento de Química, Universidad Nacional de Colombia, 111321 Bogotá, Colombia; armorenom@unal.edu.co (A.R.M.-M.); lgiraldogu@unal.edu.co (L.G.); 2 Departamento de Química, Universidad de los Andes, 111711 Bogotá, Colombia

**Keywords:** parabens, activated carbon, adsorption isotherms, immersion calorimetry, Sips

## Abstract

Parabens (alkyl-p-hidroxybenzoates) are antimicrobial preservatives used in personal care products, classified as an endocrine disruptor, so they are considered emerging contaminants. A raw version of activated carbons obtained from African palm shell (Elaeis guineensis) modified chemically by impregnation with salts of CaCl_2_ (GC2), MgCl_2_ (GM2) and Cu(NO_3_)_2_ (GCu2) at 2% *wt*/*v* and carbonized in CO_2_ atmosphere at 1173 K was prepared. The process of adsorption of methyl (MePB) and ethylparaben (EtPB) from aqueous solution on the activated carbons at 18 °C was studied and related to the interactions between the adsorbate and the adsorbent, which can be quantified through the determination of immersion enthalpies in aqueous solutions of corresponding paraben, showing the lowest-value carbon GM2, which has a surface area of 608 m^2^ × g^−1^, while the highest values correspond to the activated carbon GCu2, with a surface area of 896 m^2^ × g^−1^ and the highest content of surface acid sites (0.42 mmol × g^−1^), such as lactonic and phenolic compounds, which indicates that the adsorbate–adsorbent interactions are favored by the presence of these, with interaction enthalpies that vary between 5.72 and 51.95 J × g^−1^ for MePB adsorption and 1.24 and 52.38 J × g^−1^ for EtPB adsorption showing that the process is endothermic.

## 1. Introduction

Parabens (alkyl-p-hydroxybenzoates) are antimicrobial preservatives whose use is widely distributed, mainly in personal care products. They are referred to as emerging contaminants whose harmful effects are related to inhibition of normal hormonal system activity in animals and presumably in humans, so they are classified as an endocrine disruptor [1]. Methyl and ethylparaben are generally considered to have a much lower potential to cause endocrine disrupting effects compared to other larger parabens [2,3]. Błędzka et al. [4] in their study showed the limited accessible data on the presence of parabens in surface waters. In general, among all measured parabens, the highest frequency and concentration for methylparaben was recorded, while the presence of ethylparaben in water samples was recorded less frequently.

Currently, there are several processes that are used in the removal of endocrine disruptors; these include coagulation/flocculation [5,6], biological treatment [5,6,7], oxidation [5,6,8], and adsorption [6,9,10]. Some of these techniques are more traditional and used than others. While new methods or improvements are continually proposed, each process has advantages, disadvantages, and limitations in the removal of contaminant traces according to local conditions, which does not ensure an adequate technical–economic–environmental outcome in all cases. The particular case of adsorption on activated carbon of different organic compounds has been extensively studied or has become a viable alternative for the removal of various contaminants in water [11,12,13]. An important feature of activated carbons is their adsorption capacity, which depends on the inherent nature of the precursor, as well as the manufacturing process, which affect the textural characteristics developed in the material [14].

It is well known that activated carbons have heterogeneous surfaces. This heterogeneity is associated with a porous structure, usually with small amounts of chemically bonded heteroatoms (mainly oxygen), forming oxygen functional groups that are frequently located at the edge of the graphenic layers. Both chemical and structural heterogeneities contribute to the unique adsorption properties of activated carbons [15]. Three kinds of interactions, between the activated carbon surface and the aromatic compounds, have been mainly proposed, namely, the π–π dispersion interaction mechanism, the hydrogen bonding formation mechanism [16], and the electron donor–acceptor complex mechanism [17]. Among the large quantity of organic compounds, the adsorption of several phenols from aqueous solutions onto activated carbons has had great notoriety, and the influence of the physicochemical characteristics has been studied by many workers. Some researchers indicate that physical adsorption occurs as a result of dispersive interactions between the aromatic part of the phenol and the carbon basal planes [18], while other researchers have suggested that aromatic compounds are adsorbed on carbons through a donor–acceptor mechanism [19].

Likewise, the adsorbate–adsorbent interactions in the physical adsorption are a function of the polarity of the solid surface and the adsorbate. The apolar nature of the activated carbon surface will favor the adsorption of nonpolar molecules by a nonspecific mechanism, but the adsorption of polar molecules can be increased by an adequate modification of the chemical nature of the surface carbon, which will lead to an increase in adsorbent–surface interactions [14]. These interactions can be studied by determining the immersion enthalpy of activated charcoal in a chosen solution, since the immersion heats of a solid in various liquids are different, and this allows evaluating the specific interactions between the surface of the solid and the immersion liquid [20,21].

This work presents the results of the adsorption of two parabens, methyl and ethylparaben, in three activated carbons obtained from African palm shell by chemical activation with solutions of CaCl_2_, MgCl_2_, and Cu(NO_3_)_2_ to 2% (*wt*/*v*). Equilibrium data were evaluated from the Sips model and the adsorption capacity of activated carbon was calculated. These results were correlated to calorimetric data through the determination of immersion enthalpy in parabens solutions at two different concentrations in order to assess their effect on adsorbate–adsorbent–solvent interactions. The final goal of the work was to relate the equilibrium data and adsorbate–adsorbent interactions with textural and chemical properties of the activated carbons.

## 2. Results and Discussion

### 2.1. Activated Carbon Characterization

**Figure 1:**
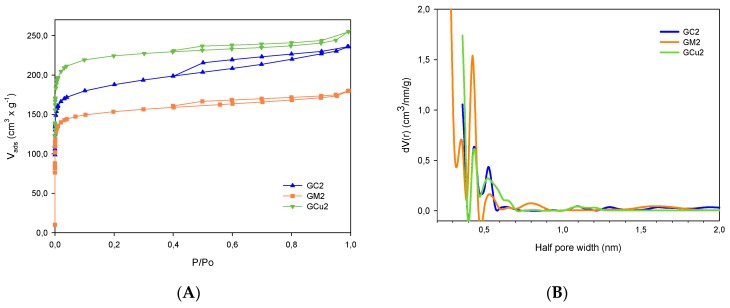
(**A**) Nitrogen adsorption isotherms of activated carbons at 77 K; (**B**) pore size distribution by the quenched solid density functional theory (QSDFT) model.

Nitrogen adsorption isotherms at 77 K are present in Figure 1A, where the modification of the textural characteristics produced by chemical impregnation treatments with metal salts is evident. Samples of GM2 and GCu2 display a typical type I adsorption isotherm, while the GC2 sample exhibits a behavior composed of isotherms type I and II according to the IUPAC classification, showing a more pronounced adsorption at low pressures relative to micropore filling, while at high pressures, capillary condensation is presented, accompanied in all isotherms by small hysteresis loops of desorption, indicating the poor development of mesoporosity in activated carbons.

The pore size distribution calculated by the density functional theory (DFT) method is shown in Figure 1B. The best fitting was obtained using the quenched solid density functional theory (QSDFT), where the interaction between the adsorbate and the adsorbent is explicitly taken into account and is considered a “rough” or “heterogeneous” surface [22,23]. For the samples studied, the best results were obtained considering a combined porous system slit/cylinder with average error percentages between 0.023% and 0.158%. In this figure, the microporous structure of activated carbons is confirmed where the average pore size is between 0.3 and 1 nm, having a greater contribution of micropores around 0.4–0.6 nm, and the almost null development of mesoporosity is confirmed. Therefore, the mesopore volume calculation usually determined by the BJH (Barrett, Joyner, and Halenda) method was omitted.

The pore structure parameters are present in Table 1, showing that impregnation with metallic salts moderately modifies the textural characteristics of activated carbons, where it is shown that, all carbons are microporous, because the microporosity corresponds practically to the total pore volume in the activated carbons. The relationship between the surface area of the carbons and the volume of micropores developed is also evident, considering that most of the total surface area is in micropores. These results can be explained by considering the smaller atomic radius that is in copper (0.128 nm), which favors the formation of microporosity, compared to those of magnesium (0.160 nm) and calcium (0.197 nm). This confirms that the reagent used in the impregnation is used as a template for the creation of porosity [24].

The results of functional group analysis of activated carbons determined by the technique proposed by Boehm are presented in Table 2. In this case, it was observed that in all cases, the treatments carried out changed the chemical characteristics of the activated carbons. In the case of total acidity, it was observed that impregnation with copper salt develops a higher content of acid icing groups on the periphery of the graphenic layers of GCu2 activated carbon produced by some oxygenated groups of carboxylic, lactonic, and phenolic, particularly the latter, because the African palm shell is constituted by lignin, cellulose, and hemicellulose [25], which are high in this group. As for total basicity, it is evident that impregnation with calcium salt favored the formation of basic groups associated with pyrone and chromene structures, as well as the presence of electrons π on basal graphene layers [26] in carbon GC2.

### 2.2. Adsorption Studies

It is important that adsorption is a very complex process, depending on the characteristics of the adsorbent as well as adsorbates, as well as affinity that is presented in said system, affecting the adsorption capacity of a given adsorbent. Table 3 presents the physicochemical properties of the parabens under study; they have low water solubility and increasing the length of the carbonated chain leads to decreased the solubility, so sodium salts are frequently used also in formulations. The values of the acid constant of dissociation (pK_a_) is around 8.2, so in aqueous solutions, they are in their protonate form.

**Figure 2:**
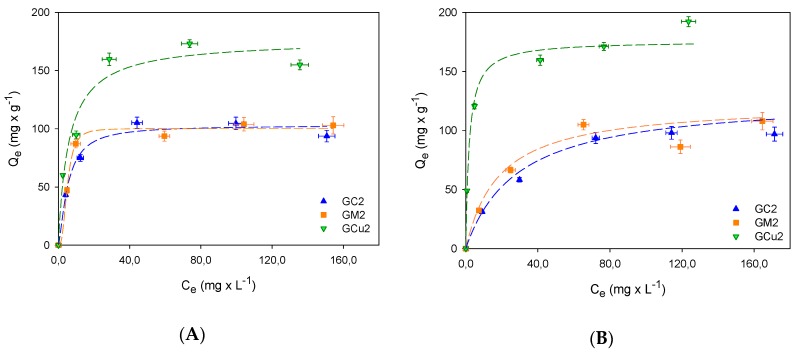
Adsorption isotherms at 18 °C. (**A**) Methylparaben; (**B**) ethylparaben.

Giles et al. [28] related the shape of the adsorption isotherms in the aqueous phase with the nature of the adsorption. According to their classification, the adsorption isotherms of methyl and ethylparaben presented in Figure 2 are identified as L-type or Langmuir isotherms, with a concave shape so that as the concentration in the liquid phase increases, the amount absorbed slowly increases.

To carry out the study of the interactions between parabens and activated carbons from an aqueous solution, adsorption data were described using the Sips model (Equation (1)). This three-parameter model is an extension of the Freundlich model that is appropriate for describing the adsorption of heterogeneous systems, but in its mathematical form, it is like the Langmuir model, which is applicable to ideal surfaces. The main difference between the Langmuir model and the Sips model is the *n_S_* parameter that describes the characteristic heterogeneity of the system derived from the adsorbent, adsorbate or a combination of both [29]:

(1) $$Q_{e}=\frac{Q_{mS}K_{S}{C_{e}}^{1/n_{S}}}{1+K_{S}{C_{e}}^{1/n_{S}}}.$$

*Q_e_* is the adsorbed amount in the equilibrium (mg × g^−1^), *C_e_* is the concentration of the adsorbate in the equilibrium (mg × L^−1^), *Q_mS_* is Sips maximum adsorption capacity of (mg × g^−1^), and *K_S_* is the Sips equilibrium constant (L × mg^−1^) for heterogeneous solids that relates to the characteristic energy of micropores.

The parameters obtained from the adjustment to the Sips model are presented in Table 4, showing that the methylparaben adsorption values are lower than those obtained for ethylparaben, as is the case for the adsorption of EtPB with the carbon GCu2 that, possessing the highest value of the constant *K_S_*, indicates that there is a greater energy interaction between EtPB with the surface of this activated carbon, which also has the highest concentration of oxygenated functional groups on its surface.

**Figure 3:**
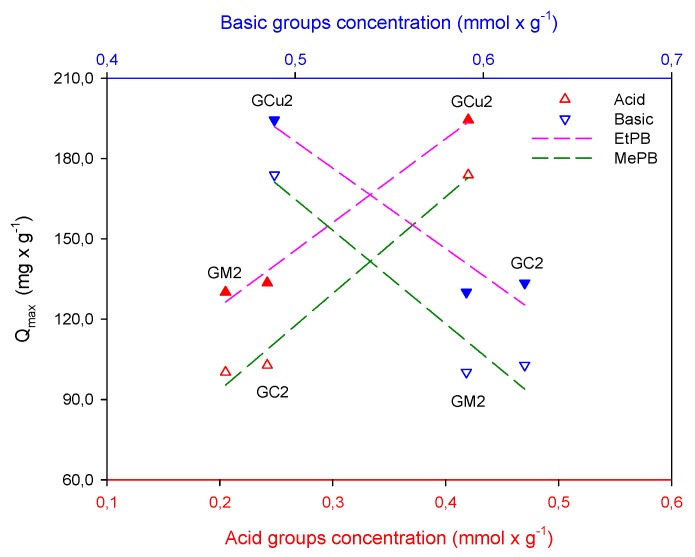
Relation between acid and basic group concentration with adsorption capacity of methyl and ethylparaben.

With respect to the *n_S_* parameter, it is indicated that the systems formed by the EtPB with the GC2 and GM2 carbons are energetically homogeneous because their value is exactly or is approximately to unity [29], while the system formed by EtPB with the GCu2 carbon by moving away from this value suggests that the system is energetically heterogeneous. As is well known, two regions are distinguished on the surface: a nonpolar and hydrophobic one that has basic properties associated with regions rich in π electrons located in the graphene basal planes and another hydrophilic region containing various functional groups [30,31]. Thus, the carbons GC2 and GM2 possess the lowest acidity and highest basicity, as shown in Figure 3, which is usually associated with π-delocalized electrons in the graphenic layers of the carbonous structure, and have dispersive hydrophobic interactions [32] with the electrons in the aromatic ring of the ethylparaben [33,34]. Carbon GCu2, meanwhile, having a higher concentration of acid groups that decrease the electron density of the basal plans of the activated carbon, favors the specific interactions that are established between these groups and the phenol present in the structure EtPB. These results suggest that in general, the presence of functional groups promotes heterogeneous interactions [35]. However, this heterogeneity of the system indicates not only adsorbent–adsorbate interactions but possible adsorbate–adsorbate associations on the surface that increase adsorption.

Similarly, the systems formed by MePB with the carbons GC2 and GM2 have an energetically homogeneous character, their value being less than the unit and for the reasons mentioned above with the EtPB, given the similarity in their structures. However, the system formed by MePB–GCu2 is also homogeneous, suggesting that a type of interaction prevails. Hence, surface chemistry may be deciding that either specific or nonspecific interaction is the prevailing mechanism for adsorption of a given adsorbate [31].

Figure 3 also shows the influence of surface chemistry on paraben adsorption, showing an almost direct interaction between the adsorbed paraben molecules and the acidic groups present on the surface, indicating that these groups are responsible for the adsorption evidenced in the different activated carbons, through the formation of specific interactions between the acidic groups on the periphery of the graphenic layers of activated carbon and the phenol groups present in paraben structures. With regard to adsorption capacities, it is shown that this is higher for EtPB, which is confirmed by various materials studied [36,37]. This is due to the decrease in solubility, as the size of the carbon chain in the ester group increases, so interaction with water is favored by decreasing the polarity of the EtPB. Therefore, paraben–adsorbent interaction will be stronger than that of paraben–solvent as the molecular size increases.

**Figure 4:**
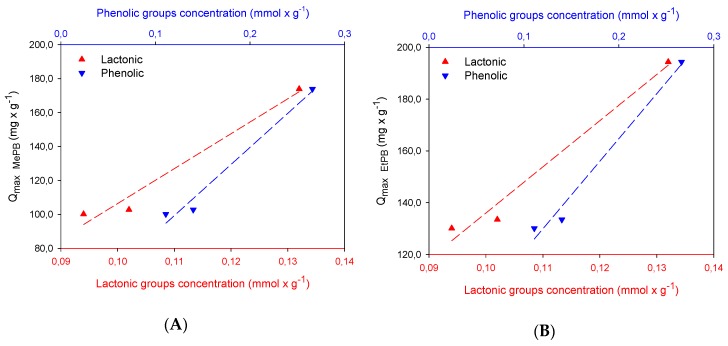
Relation between lactonic and phenolic groups with adsorption of (**A**) methylparaben and (**B**) ethylparaben.

As shown in the figure above, adsorption depends on the presence of surface groups, particularly of acid character, and expanding this information in Figure 4, it is evidenced that it depends on the interactions that are generated with phenolic and lactonic groups, through the formation of hydrogen bonds with the phenol of parabens and, on the other hand, a “donor–acceptor” complex mechanism similar to that mentioned by Mattson and co-workers [17] between the carbonyl group on the carbon surface and the aromatic ring of the solute, with the carbonyl oxygen of lactone acting as the electron donor and the aromatic ring of the paraben structure acting as the acceptor. This indicates that the paraben adsorption mechanism involves several kinds of electron-donors as carbonyl–oxygen-containing functional groups (carboxylic/lactone) on the activated carbon surface and its interaction with the paraben aromatic ring. Therefore, given the similarity in the content of lactonic groups of the GC2 and GM2 carbons, similarities are presented with the adsorption capacity between these carbons.

### 2.3. Calorimetric Study

The calorimetric study of activated carbons was carried out through the determination of immersion enthalpy in water and paraben solutions, in order to observe changes in enthalpy by concentration as well as impregnation with the different metal salts. Table 5 presents the results obtained from the immersion enthalpy, the values of which correspond to exothermic processes of the order of physisorption. Regarding water immersion enthalpy, its relationship is observed with the increase in hydrophilic functional groups at the edges of the graphenic layers, which increase the polarity of activated carbons in the order of GCu2 > GC2 > GM2.

**Figure 5:**
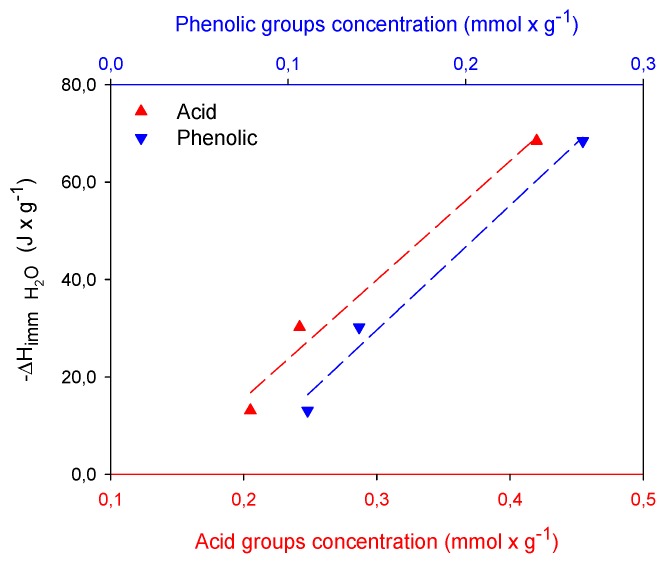
Influence of acid and phenolic group concentration over immersion enthalpy in water.

By associating these results with those of chemical characterization, it is evident that this order relates to the content of phenolic groups, as shown in Figure 5, so it suggests that adsorbent–solvent interaction is associated with the formation of a hydrogen bond between phenol and water.

**Figure 6:**
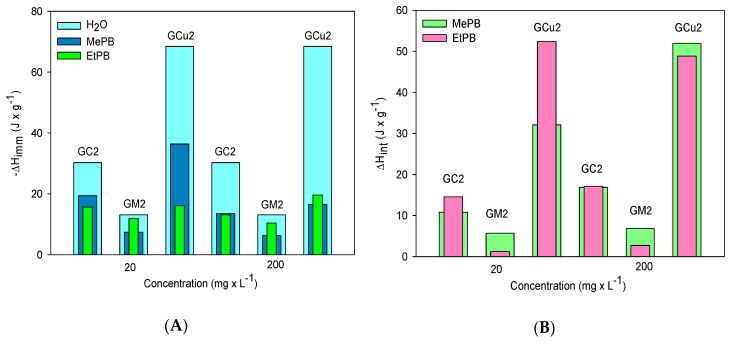
Comparation between (**A**) immersion enthalpies and (**B**) interaction enthalpies of activated carbons at different methyl and ethylparaben concentrations.

With regard to immersion enthalpy in paraben solutions, which correspond to the result of total energy produced in adsorbent–adsorbate, adsorbent–solvent, and adsorbent–solvent interactions, it is evident that it decreases with the increase in paraben concentration, suggesting that the energy transferred in the form of heat in each system decreases as the surface of the adsorbent becomes saturated [35], as shown in Figure 6A.

On the other hand, in order to evaluate the calorimetric effect of the specific interactions between parabens and activated carbons, the interaction enthalpy *(*∆*H_int_*) to both concentrations was determined, finding values from 5.72 to 51.95 J × g^−1^ for MePB and 1.24 to 52.38 J × g^−1^ for EtPB. As shown in Figure 6B, changes in the interaction enthalpy have positive values in all cases, suggesting that the process is endothermic and requires energy to be carried out; hence, it is usually associated with the breaking of interactions and water displacement by paraben molecules, which are much more apolar, and as the size of paraben molecule increases, the polarity of this decreases from the carbonaceous surface and the de-solvatation of the solute before adsorption [33]. Therefore, the EtPB–adsorbent interaction is stronger than that of MePB–adsorbent, so that π–π-type interactions are favored.

**Figure 7:**
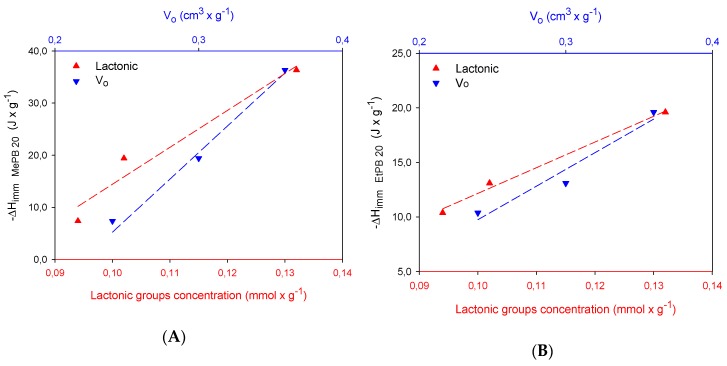
Relation between immersion enthalpy of paraben solutions at 20 mg × L^−1^ with lactonic groups and microporosity of activated carbons in (**A**) methylparaben solution and (**B**) ethylparaben solution.

Figure 7 shows the relation between the lactonic groups and the microporosity developed in activated carbons, where it is shown that the microporous structure favors interactions between parabens, because they allow the distance between the solute and the active sites on the surface of the activated carbon to be shortened, thus increasing the possibility of interactions between these, and therefore, the intensity of interactions increases. These active sites on the surface may be associated with the presence of lactonic groups, which are susceptible to hydrolysis in acidic medium, forming a carboxylic acid and an alcohol, which would favor the formation of a greater number of hydrogen bridges with the phenol of the adsorbate, while phenol formation favors that the solvent not compete for the adsorption sites created as specific solvent–phenol interactions would be formed [33]. However, although the relationship with microporosity is shown, it should be noted that given the pore size distribution (Figure 1B) of activated carbons that had a concentration of micropores in the region between 0.3 and 0.9 nm, it is suggested that there can be some diffuse restrictions if paraben molecules do not have adequate guidance to be able to enter micropores, in which case adsorption will depend more on surface chemistry.

## 3. Materials and Methods

### 3.1. Preparation of Activated Carbons

A raw version of activated carbons obtained from African palm shell (Elaeis guineensis) was modified chemically by impregnation with salts of CaCl_2_ (GC2), MgCl_2_ (GM2), and Cu(NO_3_)_2_ (GCu2) at 2% *wt*/*v* in a thermostat bath at 358 K for 48 h. The carbonization of the samples was carried out in a CARBOLITE^®^ horizontal tubular furnace (Carbolite-Gero, Hope, UK) in CO_2_ atmosphere (110 mL × min^−1^) with a 5 K × min^−1^ heating ramp for 6 h at 1173 K [38,39]. Subsequently, these were washed with HCl 5% and water to remove remain salts until the pH was constant. Finally, the activated carbons were dried in an oven for 24 h at 363 K and stored in plastic hermetically sealed containers in a nitrogen atmosphere.

### 3.2. Characterization of Activated Carbons

The textural characteristics of activated carbons were determined from N_2_ adsorption isotherms at 77 K, in an automatic equipment Autosorb 3B Quantachrome (Quantachrome Instruments, Boynton Beach, FL, USA).The apparent surface area (S_BET_) was evaluated via application of the BET model, the volume of micropore (V_o_) was calculated using the Dubinin–Radushkevich equation (D-R), and the QSDFT model was applied to nitrogen isotherms for the determination of total volume (V_T_) and the pore size distribution using ASiQWin software (Quantachrome Instruments, Boynton Beach, FL, USA, version 3.01), assuming cylinder/slit geometry over the adsorption branch.

Chemical characterization was realized using the Boehm method [30,40], with bases of different strength that allow neutralizing acids with different pKa and then determine their individual contributions. For this, 0.500 g of activated carbon was weighed and mixed with 25 mL of NaOH, HCl, Na_2_CO_3_, and NaHCO_3_ 0.05 M solutions (Merck Millipore, Bedford, MA, USA), respectively, in hermetic plastic containers. The solutions were left in agitation at 18 °C for 5 days and under 100 rpm constant agitation, with the occasional bubbling of N_2_ in order to remove atmospheric CO_2_. After some time, an aliquot of each supernatant solution was certified with the respective acid or base solutions standardized in an automatic titrator (TitroLine Alpha-Plus, Schott Instruments GnbH, Mainz, Germany). The acidic functional groups were determined from the titration of NaOH, Na_2_CO_3_, and NaHCO_3_ supernatant solutions with standardized HCl. For the quantification of the basis groups, the titration of the HCl solution with standardized NaOH solution was continued. The HCl and NaOH solutions were standardized with boric acid and potassium biphthalate, respectively. Titration experiments were performed in triplicate, and mean values were presented.

### 3.3. Adsorption Studies

Adsorption studies were carried out using 0.025 g of activated carbons prepared previously weighted on an analytical balance with an accuracy of 0.001 g (Ohaus Pioneer PA 114, Ohaus Corporation, Parsippany, NJ, USA), with 50 mL of methylparaben (MePB) and ethylparaben (EtPB) solutions, analyte grade > 99% purity (Alfa Aesar, Tewksbury, MA, USA), in a range of 20 to 200 mg × L^−1^ for 3 weeks (based on a previous kinetic study, where it was shown that the equilibrium of the system was reached after this time) at 18 °C, with sporadic agitation (100 rpm). The residual concentration of paraben was measured via UV-Vis Spectroscopy (GENESYS 10uv, spectrophotometer, Thermo Fisher Scientific, Madison, WI, USA) at 254 nm [33,37,41]:

(2) $$Q_{e}=\frac{\left(C_{o}-C_{e}\right)}{m}$$

The paraben adsorbed amount (*Q_e_*) on the activated carbons was calculated from the initial concentration (*C_o_*), equilibrium concentration (*C_e_*), paraben solution total volume (*V*), and mass of activated carbon (*m*), as presented in the balance of Equation (2).

### 3.4. Immersion Enthalpy Determination

Immersion enthalpies of activated carbons were determined, using as wet liquids water and aqueous solutions of MePB and EtPB (20 y 200 mg × L^−1^, respectively) at 18 °C on a locally built Calvet-type heat conduction microcalorimeter [42], using a stainless-steel calorimetric cell. About 0.100 g of each activated carbon was weighed into a glass vial located in the calorimetric cell containing 10 mL of the wet liquid; the electrical potential was measured for approximately 40 min until a stable baseline was obtained. The immersion of the sample and the increase in potential was recorded according to the time and product of the wet of the solid until it returned back to the baseline. The determinations were performed in triplicate, the standard deviations were calculated, and the results are shown as means values. Finally, electrical calibration was performed by providing known electrical work to the calorimetric cell [43,44]. This calibration allows calculating such electrical work (*W_elect_*), in addition to the calorimeter constant (*K_cal_*), immersion energy or heat (*E_imm_*), and finally, the immersion enthalpy (*ΔH_imm_*), as shown in Equations (3)–(6).

(3) $$W_{elect}=voltage\;\left(V\right)*current\;\left(A\right)*time\;\left(s\right)$$

(4) $$K_{cal}=\frac{W_{elect}}{Area\;under\;calibration\;peak\;}$$

(5) $$E_{imm}=\;K_{cal}*Area\;under\;immersion\;peak$$

(6) $$-\Delta H_{imm}=\frac{E_{imm}\left(J\right)}{activated\;carbon\;mass\;\left(g\right)}$$

On the other hand, the enthalpy of interaction $(\Delta H_{int}$) corresponds to energy produced by the contact between the adsorbate and the adsorbent ($\Delta H_{ads-AC}$), regardless of solvent–solvent interactions, and is determined by the Hess Law from the immersion enthalpy in paraben solutions ($\Delta H_{imm}$) and solvent–activated carbon interactions ($\Delta H_{solv-AC}$):

(7) $$\Delta H_{int}=\Delta H_{ads-AC}=\Delta H_{inm}-\Delta H_{solv-AC}.$$

Therefore, the interaction enthalpy depends not only on the adsorbent–adsorbate interactions but also on the breakdown of adsorbate–solvent and solvent–adsorbent interactions, as presented in Equation (7).

## 4. Conclusions

In this work were presented the results of the adsorption of two parabens, methyl and ethylparaben, in three activated carbons obtained from African palm shell through chemical activation with different metallic salts. The adsorption of the parabens under study can be related to the presence of acid groups, particularly lactonic groups by a complex “donor–acceptor” mechanism between the carbonyl oxygen of lactone and the aromatic ring of paraben, while the concentration of phenolic groups is more associated with the formation of hydrogen bonds with water, favoring adsorbent–solvent interactions. It was shown that both homogeneous and heterogeneous interactions may occur, due to the presence of adsorbate–adsorbent interactions, as well as adsorbent–solvent interactions. However, it was also shown that the adsorption is favored with the development of microporosity, because they allow the distance between the solute and the active sites on the surface of the activated carbon to be shortened, thus increasing the possibility of interactions, in the event that no diffuse restrictions are introduced due to the size of the solutes into the smaller pores, in which case, the surface chemistry takes on greater importance in adsorption.

The calorimetric study determined that the values of immersion enthalpy at both low and high paraben concentrations are lower than those found for the solvent (from −13.10 to −68.45 J × g^−1^), indicating the formation of solvent–adsorbent interactions and for the adsorption process to be carried out on the adsorbents, the displacement of the solvent by the adsorbate is needed, so it requires energy to carry out the rupture of the interactions with water; thus, positive interaction enthalpy values were calculated.

## Figures and Tables

**Table 1:** Pore structure parameters of activated carbons.

Samples	*S_BET_* m^2^ × g^−1^	*V_mic_* cm^3^ × g^−1^	*V_tot QSDFT_* cm^3^ × g^−1^
GC2	723	0.30	0.34
GM2	608	0.24	0.25
GCu2	896	0.36	0.35

**Table 2:** Boehm titrations results of activated carbons (mmol × g^−1^).

Samples	Carboxylic	Lactonic	Phenolic	Basic	Acidic	Total
GC2	0	0.10	0.14	0.62	0.24	0.86
GM2	0	0.094	0.11	0.59	0.20	0.80
GCu2	0.024	0.13	0.27	0.49	0.42	0.90

Standard deviation is 0.003–0.033.

**Table 3:** Physical-chemical properties of parabens [2,27].

Structures	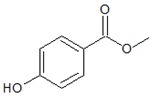	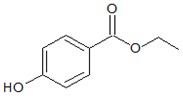
Parameters	Methylparaben (MePB)	Ethylparaben (EtPB)
Molecular weight g × mol^−1^	152.2	166.2
Molecular dimensions nm^2^	0.91 × 0.44	1.03 × 0.66
Water solubility mg × L^−1^ at 25 °C	2.50 × 10^−3^	8.85 × 10^−2^
pKa	8.17	8.22

**Table 4:** Sips model parameters of parabens adsorption.

Parameters	MePB			EtPB		
	*GC2*	*GM2*	*GCu2*	*GC2*	*GM2*	*GCu2*
*Q_mS_* mg × g^−1^	102.8	100.2	173.9	133.5	130.1	194.4
*K_S_* L × mg^−1^	0.1	0.01	0.2	0.03	0.04	0.5
*n_S_*	0.54	0.70	0.96	1.0	0.97	1.5
*r* ^2^	0.98	0.99	0.98	0.99	0.97	0.99

**Table 5:** Immersion enthalpies of activated carbons (J × g^−1^).

Samples	$-\Delta H_{imm\;H_{2}O}$	MePB		EtPB	
		$-\Delta H_{imm\;20}$	$-\Delta H_{imm\;200}$	$-\Delta H_{imm\;20}$	$-\Delta H_{imm\;200}$
GC2	30.23	19.41	13.52	15.68	13.10
GM2	13.10	7.38	6.24	11.86	10.38
GCu2	68.45	36.35	16.50	16.07	19.60

Standard deviation is 0.036–1.15.
